# Red blood cell distribution width as a predictor of mortality and poor functional outcome after acute ischemic stroke: a meta-analysis and meta-regression

**DOI:** 10.1186/s12883-024-03610-6

**Published:** 2024-04-12

**Authors:** Huiqin Shen, Lihong Shen

**Affiliations:** Department of Neurology, Huzhou Central Hospital, Affiliated Central Hospital of HuZhou University, 1558 Sanhuan North Road, Wuxing District, Huzhou City, Zhejiang Province China

**Keywords:** Stroke, Cerebral infarction, Death, Red blood cells, Recovery

## Abstract

**Background:**

This study aimed to review evidence on the ability of red cell distribution width (RDW) to predict mortality and poor functional outcomes after acute ischemic stroke (AIS).

**Methods:**

Databases of PubMed, CENTRAL, Scopus, Embase, and Web of Science were searched online from inception to 25th Jul 2023 for all studies reporting the association between RDW and outcomes as adjusted ratios. A random-effects meta-analysis was done. Meta-regression was conducted using multiple moderators.

**Results:**

15 studies with 14,968 patients were included. Meta-analysis found that RDW, both as a categorical variable (OR: 2.10 95% CI: 1.74, 2.55 I^2^ = 42%) and continuous variable OR: 1.16 95% CI: 1.05, 1.28 I^2^ = 64%) was a significant predictor of mortality after AIS. Age and number of hypertensives were found to be significant moderators in the meta-regression. Also, high RDW, as a categorical variable (OR: 1.68 95% CI: 1.20, 2.35 I^2^ = 84%), was associated with significantly higher odds of poor functional outcomes after AIS, but not as a continuous variable (OR: 1.07 95% CI: 0.99, 1.16 I^2^ = 61%). Meta-regression showed that the association was stronger in small sample-sized studies.

**Conclusion:**

RDW can be a useful, readily available, and cost-effective biomarker to rapidly stratify AIS patients at risk of poor outcomes. High RDW was consistently associated with an increased risk of mortality after AIS, however, its ability to predict poor functional outcomes needs to be verified by further studies.

**Supplementary Information:**

The online version contains supplementary material available at 10.1186/s12883-024-03610-6.

## Introduction

Stroke is amongst the most common cerebrovascular diseases diagnosed around the world. About 16.9 million individuals suffer from stroke every year causing the second-highest number of deaths and a significant proportion of disability worldwide [1]. According to estimates about 87% of the cases are due to ischemia with age being the most important risk factor [2]. Nearly 3/4 of cases of stroke occur in patients aged ≥ 65 years [3]. Given the geriatric age group, patients with acute ischemic stroke (AIS) suffer routinely from long-term complications like neurological deficits, falls, fractures, infections, etc. The patients also frequently require rehabilitation and long-term nursing care which increases the emotional and financial burden on the caregivers and the healthcare system [4]. Owing to the high burden of stroke worldwide and the associated adverse events, it is necessary to identify markers that can accurately predict the prognosis of stroke.

Red blood cell distribution width (RDW) is a commonly used hematological marker that is based on the circulating red blood cell volume. It is routinely measured by automated cell counters during blood counts and is easily available in healthcare centers across the world. The marker demonstrates the red blood cell size variation in the blood sample and is derived from the distribution curve width and the mean cell size [5]. RDW is a good prognostic marker for several diseases like atrial fibrillation, cardiac failure, ischemic heart disease, pulmonary embolism, sepsis, renal disorders, hepatic disorders, AIS, and several malignant conditions [6–12]. Given its widespread availability and low cost, RDW is being recognized as an important biomarker that can provide early primary risk stratification of patients cost-effectively even in regions with scarce healthcare resources [13].

In recent times, several studies [14–17] have examined the ability of RDW to predict mortality and poor functional outcomes- the most important outcomes of AIS. However, the results of studies have differed owing to variations in the study populations, sample size, and cut-offs of RDW. Therefore, there is a need to assimilate data from published studies to provide quality evidence. With this aim, the current review was conducted to examine if RDW can predict mortality and poor functional outcomes after AIS.

## Materials and methods

### Search strategy and inclusion criteria

Based on the recommended guidelines, we prepared the review protocol and uploaded it on the international register PROSPERO (CRD42023445726). The study was performed based on the criteria of the Preferred Reporting Items for Statistic Reviews and Meta-Analyses statement [18]. Databases of PubMed, CENTRAL, Scopus, Embase, and Web of Science were searched online from inception to 25th Jul 2023 for English-language articles. Conference proceedings and unpublished or non-peer-reviewed data were not considered during the search.

All eligible studies had to meet the following criteria: (1) Prospective or retrospective cohort studies conducted on AIS patients or reporting AIS as a subgroup of all stroke patients. (2) Reporting association between RDW and mortality or poor functional outcomes after a stroke at any follow-up period. (3) The examined association was reported as a multivariable-adjusted effect size with 95% confidence intervals (CI). (4) Used RDW as either a continuous variable or divided it into groups for the analysis (as a categorical variable).

Studies not specifically reporting on AIS, not reporting outcomes, not conducting adjusted analysis, and duplicate studies were excluded.

The search strategy was formulated using the keywords: “RDW”, “red cell distribution”, “stroke”, “cerebral ischemia”, “brain infarction”, and “cerebral infarction”. Further details are demonstrated in Supplementary Table 1.

Two reviewers were involved in the search process which first began with title and abstract screening. Studies were excluded if the title or the abstract did not conform with the aims of this review. Full text was then obtained for all identified acceptable studies, or when the relevance of an article could not be determined. Disagreements were settled by consensus. The same process was performed for the full-text review. The bibliography of included studies was cross-referenced to discover further eligible studies.

### Data extraction

Two reviewers performed the data extraction and assimilated information related to the year of the study, the author’s first name, sample size, age and gender, hypertension, diabetes, hyperlipidemia, timing of blood sample, cut-off of RDW, method of determination of cut-off, number of patients with high RDW, treatment of AIS, and follow-up. Outcome data of interest were mortality and poor functional outcome. Poor functional outcome was defined as a score of 3–6 on the modified ranking scale (mRS) [14–17].

According to the guidelines of the Newcastle-Ottawa Scale (NOS) [19], the included studies were judged for bias by two independent reviewers in the domains of selection of cohort, comparability, and outcome assessment. The three components were given points for questions included in the NOS. The total points available are: selection: 4; comparability: 2; and outcome assessment: 3.

### Statistical analysis

All extracted numerical data were expressed as absolute numbers or proportions. The effect size on the association between RDW and outcome was pooled using Odds ratio (OR) with 95% CI. Separate analyses were conducted for RDW expressed as a continuous variable or as a categorical variable. Meta-analysis was conducted in a random-effects model using the software “Review Manager” (RevMan, version 5.3). Outliners were assessed using a sensitivity analysis involving the removal of one study at a time. The chi-square-based Q statistics and I^2^ statistic was used for inter-study heterogeneity. A p-value of < 0.10 for Q statistic and I^2^ > 50% meant substantial heterogeneity.

To examine the effect of various moderators, a meta-regression analysis was performed using “metaHUN: a web tool for meta-analysis” (Available at: http://softmed.hacettepe.edu.tr/metaHUN/). The moderators selected were sample size, age, male gender, hypertension, diabetes, hyperlipidemia, and cut-off of RDW. Meta-regression was performed only for the meta-analysis based on RDW as a categorical variable (high vs. low) and not for RDW as continuous variables. This was done owing to the paucity of data for the latter analysis.

## Results

**Fig. 1:**
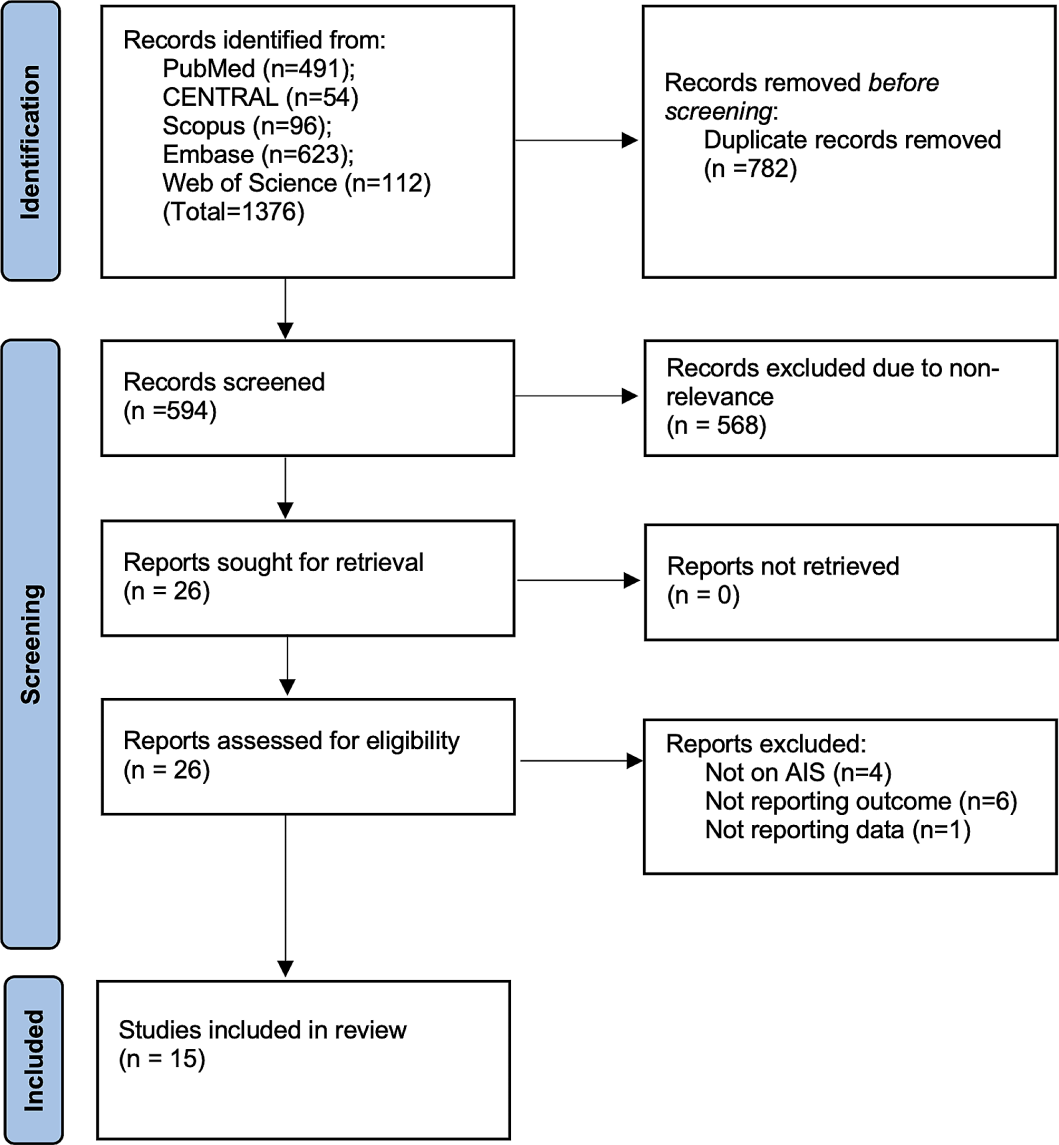
Study flowchart

The two reviewers found 1376 articles from the databases. After electronic deduplication using EndNote, 594 were screened and 26 articles were identified by the reviewers for further analysis. The inter-reviewer rating for the selection of studies was high (kappa = 0.9). Finally, based on the inclusion criteria, 15 studies were included in the review [14–17, 20–30] (Fig. 1). No additional study was found from the reference list of included studies.

Baseline study details extracted by the authors are shown in Table 1. All were retrospective cohort studies. The articles were published in 14 years between 2009 and 2023. There was a predominance of Asian studies with seven studies from China, two from Korea, and one from Taiwan. The remaining studies were from the USA, Portugal, Italy, and Turkey. The pooled sample size of all studies was 14,968. All studies included elderly patients with a mean/median age of > 60 years. In five studies, the blood samples for RDW were taken within 24 h of admission while in the remaining studies, it was on admission. Three studies used RDW only as a continuous variable. In the remaining studies, the cut-off for high RDW varied from 13 to 16%. Absolute numbers of AIS patients with high RDW were not mentioned in most studies. Similarly, treatment was also not specified in the majority of studies. The follow-up duration ranged from reporting only in-hospital outcomes to up to 75 months. The reviewers judged the studies on NOS and awarded points ranging from 6 to 9. Raw data from the included studies analyzed in the meta-analysis is presented as Supplementary Table 2.

**Table 1:** Details of included studies

Study	Location	Sample size	Male gender	Age (years)	HTN (%)	DM (%)	Current smokers (%)	HL (%)	Sample time	Cut-off of RDW	Method of determination of cut-off	% with high RDW	Treatment	Follow-up	NOS score
Ani 2009^[14]^	USA	480	228	NR	63.4	25.4	22.4	73.8	NR	13.9	Pre-determined	23.8	NR	75 months	7
Kim 2012^[15]^	Korea	847	507	65.9	72.4	29.4	24.8	21.1	On admission	NR	NR	NR	AP, IVT	3 months	7
Turcato 2017^[16]^	Italy	837	NR	77	NR	NR	NR	NR	On admission	13	ROC curve	NR	AP, IVT	3 months	8
Pinho 2018^[17]^	Portugal	602	257	60.5–80	68.4	20.8	NR	43.9	On admission	14	Pre-determined	23.1	IVT	1 year	8
Chen 2019^[25]^	Taiwan	3956	NR	NR	NR	NR	NR	NR	On admission	14.5	Pre-determined	13.6	NR	Up to 5 years	8
Gunes 2020^[26]^	Turkey	204	82	NR	78.9	34.8	NR	26.9	On admission	NR	NR	NR	NR	In-hospital	8
Wang 2020^[27]^	China	1558	838	66.2	57.5	21.1	24	7.8	Within 24 h	14	ROC curve	36.2	AP, IVT, ET	3 months	7
Ye 2020^[28]^	China	480	300	71	76.3	27.9	NR	37.7	On admission	14.6	ROC curve	NR	IVT	1 year	8
Zhao 2020^[30]^	China	2646	NR	NR	NR	NR	NR	NR	Within 24 h	14.3	Pre-determined	NR	NR	1 year	8
Akpinar 2021^[29]^	Turkey	205	110	63.7	82	31.7	17	NR	On admission	16	Pre-determined	19	ET	3 months	8
Kim 2021^[20]^	Korea	240	131	72	63.8	28.3	NR	18.3	On admission	12.8	ROC curve	NR	IVT	3 months	7
Guan 2022^[21]^	China	1576	NR	63.2	74.9	32	NR	NR	Within 24 h	NR	NR	NR	AP	1 year	7
Wang’ 2020^[22]^	China	102	57	70	51.9	13.7	10.7	8.8	On admission	13.05	ROC curve	NR	ET	1 year	8
Xue 2022^[23]^	China	629	403	70	78.4	33.2	45.3	3.5	Within 24 h	13.4	Pre-determined	23	AP, IVT, ET	3 months	9
Li 2023^[24]^	China	606	362	84.6	81	24.8	5.6	72.8	Within 24 h	13.8	Pre-determined	68.4	NR	In-hospital	7

AP, antiplatelets; IVT, intravenous thrombolysis; ET, endovascular therapy; HTN, hypertension; DM, diabetes mellitus; HL, hyperlipidemia; RDW, red cell distribution width; NOS, Newcastle Ottawa scale; NR, not reported; ROC, receiver operating characteristic

**Fig. 2:**
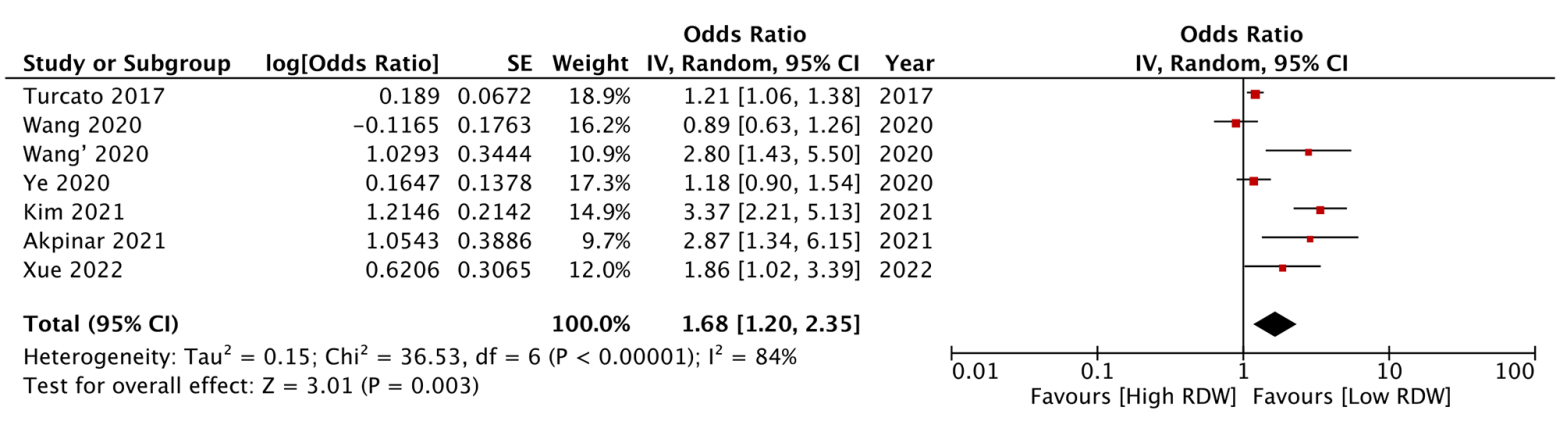
Meta-analysis of association between RDW (as categorical variable) and mortality

**Fig. 3:**
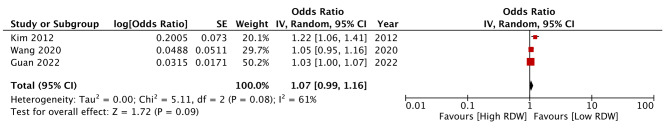
Meta-analysis of association between RDW (as continuous variable) and mortality

Examining the association between RDW as a categorical variable with a study-defined cut-off of high and low RDW, this meta-analysis noted that RDW was a significant predictor of mortality after AIS (OR: 2.10 95% CI: 1.74, 2.55 I^2^ = 42%) (Fig. 2). The review results failed to change on the exclusion of any study during the sensitivity analysis (results are not shown). Meta-regression outcomes are shown in Table 2. A significant positive association was noted for the moderators’ age and hypertensives. Higher age and percentage of hypertensives were associated with a stronger association between RDW and mortality. Only five studies used RDW as a continuous variable. Pooled analysis showed a significant association between the incremental increase of RDW and mortality after AIS (OR: 1.16 95% CI: 1.05, 1.28 I^2^ = 64%) (Fig. 3). Results remained significant on sensitivity analysis (results are not shown).

**Table 2:** Meta-regression for mortality with RDW as a categorical variable

Moderator	Effect Size	Standard Error	Lower CI	Upper CI	Z Score	P Value
Sample size	-0.0004	0.0002	-0.0008	0.0001	-1.6472	0.0995
Male gender	0.0009	0.0021	-0.0032	0.0050	0.4278	0.6688
Age	0.1746	0.0599	0.0572	0.2921	2.9138	0.0036
HTN	0.1105	0.0403	0.0315	0.1896	2.7396	0.0062
DM	0.1168	0.1115	-0.1017	0.3353	1.0479	0.2947
HL	0.0283	0.0195	-0.0099	0.0666	1.4514	0.1467
Cut off of RDW	-0.3320	0.6603	-1.6260	0.9621	-0.5028	0.6151

CI, confidence interval; HTN, hypertension; DM, diabetes mellitus; HL, hyperlipidemia; RDW, red cell distribution width

**Fig. 4:**
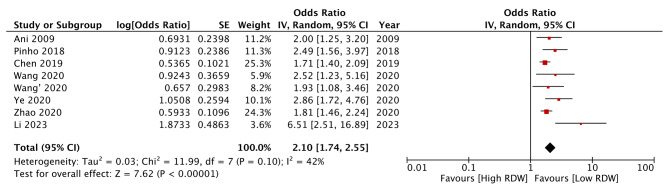
Meta-analysis of association between RDW (as categorical variable) and poor functional outcomes

**Fig. 5:**
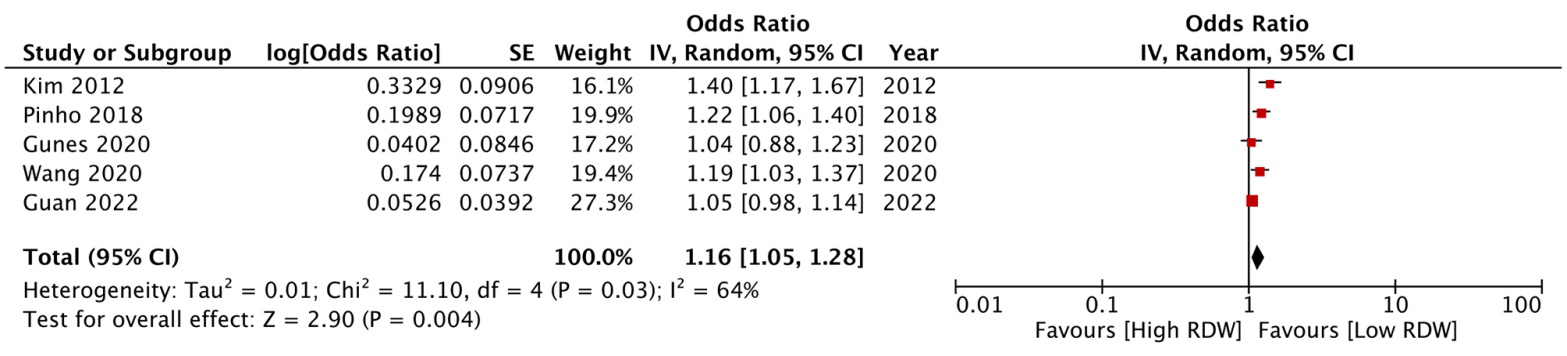
Meta-analysis of association between RDW (as continuous variable) and poor functional outcomes

Using RDW as a categorical variable with a study-defined cut-off of high and low RDW, we found that high RDW was associated with significantly higher odds of poor functional outcomes after AIS (OR: 1.68 95% CI: 1.20, 2.35 I^2^ = 84%) (Fig. 4). The effect size did not lose its significance on sensitivity analysis (results are not shown). Outcomes of meta-regression are shown in Table 3. Except for the sample size, none of the moderators had a significant association with the effect size. It was noted that studies with lower sample sizes found a stronger association between RDW and poor functional outcomes. Only three studies used RDW as a continuous variable for examining poor functional outcomes. The meta-analysis did not find a significant association between the incremental increase of RDW and poor functional outcomes after AIS (OR: 1.07 95% CI: 0.99, 1.16 I^2^ = 61%) (Fig. 5).

**Table 3:** Meta-regression for functional outcome with RDW as a categorical variable

Moderator	Effect Size	Standard Error	Lower CI	Upper CI	Z Score	P Value
Sample size	-0.0015	0.0006	-0.0026	-0.0003	-2.5492	0.0108
Male gender	-0.0028	0.0016	-0.0058	0.0003	-1.7686	0.0770
Age	0.0204	0.1372	-0.2485	0.2893	0.1487	0.8818
HTN	-0.0277	0.0574	-0.1402	0.0847	-0.4837	0.6286
DM	-0.0147	0.0818	-0.1750	0.1456	-0.1795	0.8576
HL	-0.0127	0.0510	-0.1126	0.0872	-0.2490	0.8033
Cut off of RDW	-0.9707	0.6408	-2.2267	0.2853	-1.5147	0.1298

CI, confidence interval; HTN, hypertension; DM, diabetes mellitus; HL, hyperlipidemia; RDW, red cell distribution width

## Discussion

AIS is a leading cause of mortality around the world but there still exists a deficiency of simple biomarkers with can rapidly identify high-risk populations. Biomarkers are important for initial screening and prioritization of treatment to reduce the risk of adverse outcomes. In this context, RDW is a potential biomarker that is part of routine blood counts and can be examined dynamically in a cost-effective manner. Higher RDW means reduced deformability of the erythrocyte indicating damage to the microcirculatory blood flow which reduces tissue level oxygen supply [6]. Indeed, several studies have demonstrated a strong association between RDW and poor outcomes in multiple diseases [6–12]. Focusing on cerebrovascular diseases, high RDW has been associated with an increased risk of cerebral infarction and poor outcomes after aneurysmal subarachnoid hemorrhage [31]. RDW also predicts 30-day mortality after traumatic brain injury [32]. Lorente et al [33] have shown that RDW can be a good biomarker to predict mortality after spontaneous intracerebral hemorrhage. A previous meta-analysis has found that high RDW in the general population increases the risk of stroke occurrence by 53% [34]. In the same study, the authors also evaluated the prognostic ability of RDW for stroke but could include just eight studies. Furthermore, the study combined both crude and adjusted associations between RDW and outcomes.

The current review consolidated data from 15 studies in the literature to examine the prognostic ability of RDW for mortality and poor functional outcomes after AIS. We pooled only adjusted data from studies to reduce the risk of confounding. Owing to differences in the presentation of data, a separate analysis was conducted for studies using RDW as a continuous or categorical variable. In the first part of the meta-analysis, pooled data from eight studies showed that high RDW (ranging from 13 to 14.6%) was associated with a statistically significant 2.1 times increased risk of mortality as compared to those with lower RDW at or within 24 h of admission. Scrutiny of the forest plot revealed consistency of outcomes amongst studies for such association albeit with a difference in overall effect size. Individual studies found a 1.7 to 6.5 times increased risk of mortality amongst those with high RDW. On the other hand, when RDW was used as a continuous variable, the effect was subdued with pooled analysis showing only a 16% increased risk of mortality. These results show that when used as a categorical variable, RDW in the range of 13 to 14.6% is associated with significantly increased risk of mortality. Also, per unit increase in RDW is also associated with increased mortality. Given the baseline differences amongst studies for several variables which can modify survival after AIS, a meta-regression analysis was conducted. It was found that studies including more elderly patients and a higher number of hypertensives reported a stronger association between RDW and mortality. It is well known that the number of comorbidities increases with age. Also, the elderly have lower medicine compliance, altered metabolism, and higher drug interactions with polypharmacy which make them a unique risk group [35]. Research has shown that older age is a significant risk factor for mortality after AIS [36]. Likewise, elevated baseline blood pressure has been associated with higher clot burden, reduced chances of recanalization and good functional outcomes, increased infarct volumes, higher mortality, and early AIS recurrence [37, 38].

In the second part of the meta-analysis, we examined the relationship between RDW and poor functional outcomes after AIS only to find that patients with high RDW had a statistically significant 68% increased risk of poor functional outcomes. Unlike mortality outcome, the results of individual studies differed for this variable with two of the six studies demonstrating no significant prognostic role of AIS for poor functional outcomes. Scarce data with RDW as a continuous variable also demonstrated a non-significant effect. Importantly, the meta-regression analysis which examined the effect of several moderators for any influence on the pooled effect size showed a significant relationship for sample size. Studies with smaller sample sizes showed a stronger association between RDW and poor functional outcomes as compared to larger studies. This is a classic example of small study effects noted in the literature, wherein the effect size of smaller studies is often large due to publication bias. Smaller studies with significant results tend to be published irrespective of their quality and even there is a bias among authors to report significant results [39].

The mechanism between high RDW and AIS outcomes is still unclear but several explanations have been put forward. Inflammation has an important role in the pathophysiology of AIS with higher levels of inflammatory markers predicting poor functional outcomes and mortality after stroke [40]. High RDW may reflect higher baseline systemic inflammation since inflammatory cytokines can inhibit the bone marrow and delay erythropoietin-induced erythrocyte maturation [41]. Higher levels of C-reactive protein, interleukins, and tumor necrosis factor-alpha have been associated with higher RDW [42]. High oxidative stress levels have also been linked with anisocytosis and higher RDW [43]. Inflammation and oxidative stress can cause the development of atherosclerosis and research shows that RDW is positively associated with intima-media thickness of the carotid arteries [44]. Higher RDW also indicates a dysregulated state of erythrocyte homeostasis and impaired red blood cell production which can reduce tissue oxygenation after AIS contributing to delayed healing and poor outcomes [6].

The limitations of this review need to be commented on. Data used in this study is primarily from observational studies which have some inherent biases. Secondly, the study cohorts differed on multiple counts like baseline AIS severity, comorbidity levels, treatment protocols, and follow-up. It was unclear what medications were the patients prescribed at the time of sample collection and what were the erythrocyte sedimentation rate values. These differences could be the primary drivers of high heterogeneity noted in the meta-analyses. To partially overcome such variations, we used only adjusted data in the meta-analysis and conducted a meta-regression analysis to look for confounders. However, it is plausible that many unknown confounders could have been missed by the studies and affected the results. Thirdly, the variable cut-offs of RDW were a significant drawback. The meta-analysis showed the prognostic value of RDW for poor outcomes after AIS but could not suggest the optimal cut-off to identify high-risk patients. Lastly, the predominance of Asian and especially Chinese studies restricts the applicability of results to the global population.

To conclude, RDW can be a useful, readily available, and cost-effective biomarker to rapidly stratify AIS patients at risk of poor outcomes. High RDW was consistently associated with an increased risk of mortality after AIS, however, its ability to predict poor functional outcomes needs to be verified by further studies. Future studies should be of large sample sizes, from different geographical regions, and use similar cut-offs for better assessment of RDW as a biomarker for AIS.

### Electronic supplementary material

Below is the link to the electronic supplementary material.

Supplementary Material 1

Supplementary Material 2

Supplementary Material 3

## Data Availability

The authors confirm that the data supporting the findings of this study are available within the article and its supplementary materials.
