# Supplementary material for: Red blood cell distribution width as a predictor of mortality and poor functional outcome after acute ischemic stroke: a meta-analysis and meta-regression
Source: BMC Neurol. 2024 Apr 12;24:122. doi: 10.1186/s12883-024-03610-6 (PMC11010342; doi:10.1186/s12883-024-03610-6)
Supplement: Supplementary file 1 — Supplementary Material 1 [file 12883_2024_3610_MOESM1_ESM.docx]

Supplementary Table 1: Query and search details

| Query | Search Details |
| --- | --- |
| ((RDW) OR (red cell distribution)) AND (cerebral ischemia) | ("RDW"[All Fields] OR (("erythrocytes"[MeSH Terms] OR "erythrocytes"[All Fields] OR ("red"[All Fields] AND "cell"[All Fields]) OR "red cell"[All Fields]) AND ("distribute"[All Fields] OR "distributed"[All Fields] OR "distributer"[All Fields] OR "distributers"[All Fields] OR "distributes"[All Fields] OR "distributing"[All Fields] OR "distributional"[All Fields] OR "distributions"[All Fields] OR "supply and distribution"[MeSH Subheading] OR ("supply"[All Fields] AND "distribution"[All Fields]) OR "supply and distribution"[All Fields] OR "distribution"[All Fields]))) AND ("cerebral ischaemia"[All Fields] OR "cerebral infarction"[MeSH Terms] OR ("cerebral"[All Fields] AND "infarction"[All Fields]) OR "cerebral infarction"[All Fields] OR ("cerebral"[All Fields] AND "ischemia"[All Fields]) OR "cerebral ischemia"[All Fields] OR "brain ischemia"[MeSH Terms] OR ("brain"[All Fields] AND "ischemia"[All Fields]) OR "brain ischemia"[All Fields]) |
| ((RDW) OR (red cell distribution)) AND (brain infarction) | ("RDW"[All Fields] OR (("erythrocytes"[MeSH Terms] OR "erythrocytes"[All Fields] OR ("red"[All Fields] AND "cell"[All Fields]) OR "red cell"[All Fields]) AND ("distribute"[All Fields] OR "distributed"[All Fields] OR "distributer"[All Fields] OR "distributers"[All Fields] OR "distributes"[All Fields] OR "distributing"[All Fields] OR "distributional"[All Fields] OR "distributions"[All Fields] OR "supply and distribution"[MeSH Subheading] OR ("supply"[All Fields] AND "distribution"[All Fields]) OR "supply and distribution"[All Fields] OR "distribution"[All Fields]))) AND ("brain infarction"[MeSH Terms] OR ("brain"[All Fields] AND "infarction"[All Fields]) OR "brain infarction"[All Fields]) |
| ((RDW) OR (red cell distribution)) AND (cerebral infarction) | ("RDW"[All Fields] OR (("erythrocytes"[MeSH Terms] OR "erythrocytes"[All Fields] OR ("red"[All Fields] AND "cell"[All Fields]) OR "red cell"[All Fields]) AND ("distribute"[All Fields] OR "distributed"[All Fields] OR "distributer"[All Fields] OR "distributers"[All Fields] OR "distributes"[All Fields] OR "distributing"[All Fields] OR "distributional"[All Fields] OR "distributions"[All Fields] OR "supply and distribution"[MeSH Subheading] OR ("supply"[All Fields] AND "distribution"[All Fields]) OR "supply and distribution"[All Fields] OR "distribution"[All Fields]))) AND ("cerebral infarction"[MeSH Terms] OR ("cerebral"[All Fields] AND "infarction"[All Fields]) OR "cerebral infarction"[All Fields]) |
| ((RDW) OR (red cell distribution)) AND (stroke) | ("RDW"[All Fields] OR (("erythrocytes"[MeSH Terms] OR "erythrocytes"[All Fields] OR ("red"[All Fields] AND "cell"[All Fields]) OR "red cell"[All Fields]) AND ("distribute"[All Fields] OR "distributed"[All Fields] OR "distributer"[All Fields] OR "distributers"[All Fields] OR "distributes"[All Fields] OR "distributing"[All Fields] OR "distributional"[All Fields] OR "distributions"[All Fields] OR "supply and distribution"[MeSH Subheading] OR ("supply"[All Fields] AND "distribution"[All Fields]) OR "supply and distribution"[All Fields] OR "distribution"[All Fields]))) AND ("stroke"[MeSH Terms] OR "stroke"[All Fields] OR "strokes"[All Fields] OR "stroke s"[All Fields]) |
