# Supplementary material for: Red blood cell distribution width as a predictor of mortality and poor functional outcome after acute ischemic stroke: a meta-analysis and meta-regression
Source: BMC Neurol. 2024 Apr 12;24:122. doi: 10.1186/s12883-024-03610-6 (PMC11010342; doi:10.1186/s12883-024-03610-6)
Supplement: Supplementary file 2 — Supplementary Material 2 [file 12883_2024_3610_MOESM2_ESM.docx]

Supplementary Table 2: Outcome data from individual studies

| Study | RDW as categorical variable (high vs low) | | RDW as a continuous variable | |
| --- | --- | --- | --- | --- |
| Ani 2009^[14]^ | Mortality | Poor functional outcomes | Mortality | Poor functional outcomes |
| Kim 2012^[15]^ | 2 (1.25,3.20) |  |  |  |
| Turcato 2017^[16]^ |  |  | 1.40 (1.17, 1.67) | 1.22 (1.06, 1.41) |
| Pinho 2018^[17]^ |  | 1.21 (1.06, 1.38) |  |  |
| Chen 2019^[25]^ | 2.49 (1.56, 3.97) |  | 1.22 (1.06, 1.40) |  |
| Gunes 2020^[26]^ | 1.71 (1.40, 2.09) |  |  |  |
| Wang 2020^[27]^ |  |  | 1.04 (0.88, 1.23) |  |
| Ye 2020^[28]^ | 2.52 (1.23, 5.16) | 0.89 (0.63, 1.26) | 1.19 (1.03, 1.37) | 1.05 (0.95, 1.16) |
| Zhao 2020^[30]^ | 2.86 (1.72, 4.76) | 1.18 (0.90, 1.54) |  |  |
| Akpinar 2021^[29]^ | 1.81 (1.46, 2.24) |  |  |  |
| Kim 2021^[20]^ |  | 3.37 (2.21, 5.13) |  |  |
| Guan 2022^[21]^ |  |  | 1.05 (0.98, 1.14) | 1.03 (1, 1.07) |
| Wang’ 2020^[22]^ | 1.93 (1.08, 3.46) | 2.80 (1.43, 5.50) |  |  |
| Xue 2022^[23]^ |  | 1.86 (1.02, 3.39) |  |  |
| Li 2023^[24]^ | 6.51 (2.51, 16.89) |  |  |  |

Figures in parenthesis indicate 95% confidence intervals
